# Functional Piezoresistive Polymer Composites Based on CO_2_ Laser-Irradiated Graphene Oxide-Loaded Polyurethane: Morphology, Structure, Electrical and Piezoresistive Properties

**DOI:** 10.3390/nano13010168

**Published:** 2022-12-30

**Authors:** Chiara Mastropasqua, Antonino Veca, Alessandro Damin, Valentina Brunella, Federico Cesano

**Affiliations:** 1 Department of Chemistry and NIS (Nanostructured Interfaces and Surfaces) Interdepartmental Centre, University of Torino & INSTM-UdR Torino, Via P. Giuria 7, 10125 Torino, Italy; 2 C.R.F. S.C.p.A.—Stellantis—Materials & Sustainability Engineering, C.so Settembrini 40, 10135 Torino, Italy

**Keywords:** GO, polyurethane leather, laser irradiation, conductive wires, piezoresistive properties, morphology, structure, DC and AC transport properties

## Abstract

Nanocomposite materials have recently attracted great attention for their wide range of applications, such as in smart materials, flexible electronics, and deformation sensing applications. Such materials make it possible to combine a polymer with functional fillers. In this study, flexible artificial leathers, exhibiting insulating properties and containing 1.5 or 2wt.% of graphene oxide (GO) in the polyurethane (PU) layer, were electrically activated via CO_2_ laser irradiation to obtain conductive paths at the surface exposed to the laser beam. As the material retained its insulating properties out of the irradiation areas, the laser scribing method allowed, at least in principle, a printed circuit to be easily and quickly fabricated. Combining a variety of investigation methods, including scanning electron microscopy (SEM), optical profilometry, IR and Raman spectroscopies, and direct current (DC) and alternate current (AC) electrical measurements, the effects of the laser irradiation were investigated, and the so-obtained electrical properties of laser-activated GO/PU regions were elucidated to unveil their potential use in both static and dynamic mechanical conditions. In more detail, it was shown that under appropriate CO_2_ laser irradiation, GO sheets into the GO/PU layer were locally photoreduced to form reduced-GO (RGO) sheets. It was verified that the RGO sheets were entangled, forming an accumulation path on the surface directly exposed to the laser beam. As the laser process was performed along regular paths, these RGO sheets formed electrically conductive wires, which exhibited piezoresistive properties when exposed to mechanical deformations. It was also verified that such piezoresistive paths showed good reproducibility when subjected to small flexural stresses during cyclic testing conditions. In brief, laser-activated GO/PU artificial leathers may represent a new generation of metal-free materials for electrical transport applications of low-current signals and embedded deformation sensors.

## 1. Introduction

The field of polymer nanocomposites has attracted great scientific and industrial interest due to the numerous improvements achieved in the last few decades. The increased properties of these materials, including flame resistance [1], gas barrier [2], mechanical strength/stiffness [3], and electrical/thermal conductivity [4,5], are influenced by the impressive combination of the polymer with one or more fillers and additives [6,7]. Electrical conductivity in insulating polymers can be typically obtained by mixing the polymer with conductive fillers or by blending it with intrinsic conductive polymers [8,9]. Among all polymers, thermoset polyurethane (PU) is tailored to suit a wide variety of applications, from soft and flexible foams to tough and hard materials, depending on the native precursors (i.e., isocyanate and polyol types) and how they are formulated and cured. For example, polyol has long and flexible segments, making it a soft and elastic polymer, while tougher or more rigid polymers are formed with a higher amount of crosslinking [10]. The main innovations in polyurethane composites come from the use of natural oils [11] and/or the addition of nanofillers. Beyond typical fillers with micrometric dimensions having different spatial orientations and dispersion degrees at the microscale, nanofillers may result in more homogeneous distribution in the polymer matrix at the nanoscale with a higher interfacial contact area, thus allowing the most effective interactions between matrix and nanoparticles [12]. Nanocarbons are an obvious choice for reinforcing polymer matrices and simultaneously providing electrical and thermal conductivity to composite materials [13]. Within carbon nanofillers, graphene oxide (GO) is a well-known graphene derivative. GO is represented by sp^3^ hybridized carbon atoms and oxygen functional groups (i.e., hydroxyl and epoxy groups on the basal plane of sheets and carboxyl groups at their edges) in the Lerf–Klinovski model [14,15]. According to this model, a GO sheet contains two kinds of regions:  aromatic regions with unoxidized benzene rings and six-membered ring aliphatic regions. However, the presence of polar functionalities by interrupting the aromatic domains makes GO hydrophilic and an electrical insulator [16]. Interestingly, GO nanosheets can be finely dispersed in polar solvents [17]. More interestingly, GO can be thermally or chemically reduced (RGO) to reconstruct the hexagonal lattice for restoring graphene’s properties, including electrical conductivity [18,19]. 

The addition of graphene, its derivatives, or other nanocarbons into polymers to form polymer composites with ‘*localized*’ properties constitutes an even more advanced field of innovation for the development of material-integrated sensors, whose functions (e.g., electrical and/or sensor properties) are embedded in the composite materials [20,21,22,23]. Among the most effective methods for undertaking localized processes on composite materials is laser processing. For example, CO_2_ laser processing of polymers was initially used to cut composites via high beam power (c.a. >100–300 W) [24]. More recently, the CO_2_ laser scribing process was adopted to electrically functionalize polymers under moderate laser power (2–5 W) at the surface of polyimide (PI) without the presence of carbon nanofillers [25]. The authors reported the locally photothermal conversion of PI into graphene (i.e., LIG, laser-induced graphene) for the fabrication of supercapacitors at the surface of the polymer substrate. The fabrication of electrically percolating metal-free paths, thus retaining the insulating properties outside the irradiation area, was also explored for carbon-nanotube-based polymer composites for a laser power of c.a. 10–50 W [20,26,27]. Actually, the filamentous structure of carbon nanotubes that appear ideal for making percolative paths has revealed a few drawbacks, including production costs and environmental and toxicity issues that have undoubtedly limited their use to this end. Other polymers and nanocarbons, including nanographite and graphene, have also been explored, but the main constraint of carbon wires is an electrical resistance as high as around 1 kΩ per cm of track [28] or smaller (c.a. 0.1–0.01 kΩ per cm of wire) [29]. 

In this work, the laser activation of the GO-filled PU leather surface to fabricate electrically activated paths is reported for the first time. It will be shown that the effect of laser is threefold: (i) photothermal reduction in the GO sheets of the outer PU leather layer due to the strong interaction of GO sheets with the laser beam [19]; (ii) fabrication of electrically conductive RGO paths inside an insulating matrix due to entangled RGO sheets forming a continuous accumulation region when exposed to laser irradiation; and (iii) testing of piezoresistive properties under cyclic flexural operations of laser-activated regions. The results of this work pave the way for a wide range of new applications of PU-based leathers, such as smart materials, flexible embedded electronics, and strain-sensing applications [30,31,32,33]. 

## 2. Materials and Methods

Synthetic leather samples with the GO/PU nanocomposite layer were supplied by Nanesa Srl and SPAC SpA. GO/PU composite leathers were treated using CO_2_ laser equipment (FlyCO_2_/Towermark XL laser engraving machine, Lasit, Torre Annunziata (NA), Italy), consisting of a 10,6 μm wavelength laser beam, equipped with a scanning head with focusing optics and 150 mm focal length (Lasit, Torre Annunziata (NA), Italy). Electromagnetic emission with a maximum power of 100 W occurred in pulsed mode (PWM type), at 5–15 kHz, generating a laser spot size of about 300 μm. Laser treatments were conducted in static air, compressed air, or under N_2_ gas flow conditions. Before each process, the focal lens was carefully cleaned with a cloth soaked in acetone. Operations were carried out by varying the following process parameters: power (P), frequency (F) in the 5–15 kHz range, writing speed (v), and repetition number (N), which defines the number of irradiations along the same path.

The sample morphology and texture were investigated by means of an Evo50 SEM (Zeiss, Oberkochen, Germany) equipped with an energy-dispersive X-ray (EDX) detector. Morphological investigation was performed at an acceleration potential as low as 5–10 keV. Samples were cryo-fractured to obtain cross-sections. Profilometry analysis was performed using the Leica DCM81 confocal profilometer (Wetzlar, Germany) to evaluate the 3D morphology of the conductive paths.

Thermogravimetric (TGA) analyses were performed by means of TA Q500 instrument (TA Instruments, New Castle, DE, USA) from 20 °C to 700 °C (heating rate: 10 °C/min), with an inert gas of nitrogen (flow rate of 40 mL/min), and then from a temperature of 700 °C to 800 °C in air (flow rate of 60 mL/min). This method was adopted to verify polymer thermal resistance and carbon contents. 

IR and Raman spectra were acquired on unperturbed and on laser-treated samples through a Bruker Invenio (Billerica, MA, USA) FTIR spectrometer equipped with an ATR unit and In Via Raman spectrometer (Renishaw plc, Wotton-under-Edge, UK) equipped with a 20× objective and 244 nm and 785 nm laser lines. Laser power during the Raman acquisitions was minimized (i.e., 1% or 5%) to avoid damaging the sample.

DC electrical measurements on GO/PU leathers before and after processing were performed using both two-probe and four-probe methods connected with a Keithley 2420 source meter (Keithley Instruments, Solon, OH, USA). Ag paste was used to make electrodes, which were connected with Cu wires working as connecting leads. Impedance properties were measured by means of a potentiostatic electrochemical impedance spectroscopy (EIS) SP-150 potentiostat (BioLogic Science Instruments, Vaucanson, France) in the range of 1 Hz–900 kHz at 10 mV oscillation. The EIS Spectrum Analyzer 1.0 package (Minsk, Belarus) [34] was used for impedance data interpretation and for extracting the equivalent circuit models.

The piezoresistive properties of laser-irradiated regions were obtained under a three-point bending flexural test of samples induced by an Instron 5544 Series dynamometer (Instron, Norwood, MA, USA). The instrument allowed making repeated cycles of flexural tests (N = 100, 500 and 1000) with different upward and downward speeds (50 and 100 mm/s) and with maximum deflections at the center of the samples (4 mm or 5 mm). The corresponding changes in resistance (R) values were monitored by means of a Keithley 2700E digital multimeter (Keithley Instruments, Solon, OH, USA).

Aging tests were carried out in the climatic chamber (ACS-Angelatoni Challenge 600 Environmental Chamber, Angelantoni, Massa Martana, Italy). Three tracks of the sample GO/PU were spaced out to avoid possible contact between them and connected with Cu wires. Thermal treatment from 20 to 80 °C (heating rate = 1 °C/min) was carried out. 

## 3. Results and Discussion

### 3.1. Morphology, Structure, and Thermal Analysis of GO/PU-Based Leather

**Figure 1:**
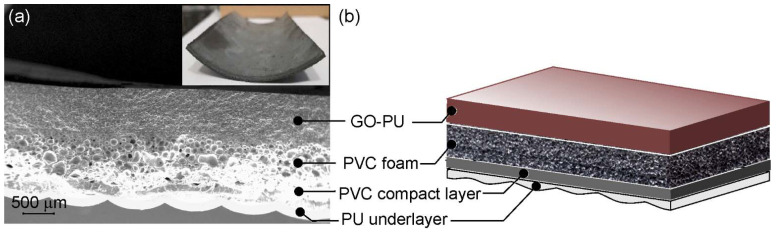
Multilayered leather: (**a**) SEM cross-sectional view; (**b**) representation of the layer structure and composition. In the inset of (**a**), a picture of flexible layered leather is shown.

The multilayered structure of the leather is shown in Figure 1. The outermost layer consisted of a GO (1.5 or 2 wt.%)-loaded PU layer c.a. 600 μm and two different PVC layers, one foamed and one more compact c.a. 800 and 200 μm in thickness, respectively. 

Finally, underneath the compact PVC layer, there was a PU underlayer of variable thickness for the good appearance and touching of synthetic leather. The structure and composition of the different layers had a soft texture; the material is flexible and soft to the touch, suitable for covering surfaces. This complex structure represents a good material for housing integrated electronics [30]. 

**Figure 2:**
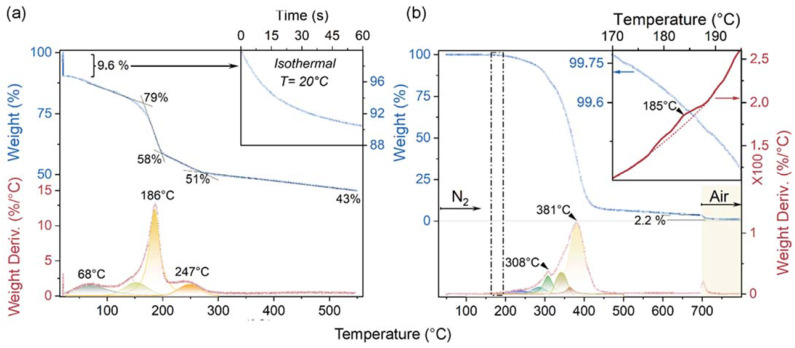
TGA (blue curves) and DTGA (red curves) plots of GO sheets (**a**) and 2 wt.% GO-loaded PU leather layer (**b**), obtained under N_2_ (up to 700 °C) or air flow (from 700 °C up to 800 °C). In the insets of (**a**,**b**), the isothermal treatment of GO at 20 °C under N_2_ gas flow and the thermograms zoomed in to the 170–195 °C interval for the GO-loaded PU leather layer are shown, respectively.

TGA/DTGA analyses of the GO and 2 wt.% GO-loaded PU layer are shown in Figure 2a and b, respectively. 

The TGA plot of GO sheets thermally treated under inert conditions (Figure 2a, blue dotted curve) showed weight loss occurring from room temperature to 550 °C. Upon isothermal treatment at 20.1 °C for 60 min (inset of Figure 2a), weight loss of about 10% was observed. In addition, residual weight for GO was 79, 58, 51 and 43 wt.% at 165, 196, 250, and 550 °C, respectively. From the related DTGA weight loss derivative (Figure 2a, red dotted curve), maxima at 68, 186, and at 247 °C were observed. It is noteworthy that weight loss occurring at lower temperatures (< c.a. 120 °C) mainly refers to physiosorbed water on the GO surface, while the remarkable weight stepdown up to 200 °C, matching a maximum in the DTGA plot centered at 186 °C, can be attributed to the decomposition of labile oxygen functional groups (i.e., hydroxyl and carboxylic acid groups) occurring during thermal reduction in GO [35]. The TGA plot of the 2 wt.% GO-loaded PU layer (Figure 2b, blue-dotted curve) showed an apparently sharp TGA profile, with a remarkable loss in weight from about 200 °C up to 440 °C (with several convoluted DTGA bands forming maxima at 308 and 381 °C, as shown in Figure 2b, red-dotted curve). The band envelope was assigned to the decomposition of urethane bonds and of ester groups occurring at lower (200–350 °C) and higher (350–450 °C) temperatures [36], respectively. At higher temperatures, the DTGA plot was more linear up to 700 °C. At 700 °C, a second remarkable loss in weight was observed after switching from nitrogen to air flow. This high-temperature weight loss (2,2 wt.%) can be attributed to the combustion of the formed RGO nanosheets. A deeper investigation of the DTGA signal (inset of Figure 2b, red curve) revealed a very broad signal with a maximum centered at 185 °C, corresponding to the weight loss also coming from thermal reduction in GO. 

In conclusion, from the TGA experiments, it was possible to observe that thermal reduction in GO sheets also formed RGOs in the GO-loaded PU layer. The residual content (c.a. 2.2 wt.%), obtained from the TGA plot at 800 °C is very close to the value of GO in the PU layer (i.e., 2 wt.%), with the contribution of char residues coming from the PU aromatic domains. It will be shown (see ATR spectra) that aromatic domains in PU are low in quantity. 

### 3.2. CO_2_ Laser Treatments on the GO-Loaded PU Layer

It is known that the interaction of laser with carbon-filler-loaded polymers (CNTs, graphene, GNPs, GOs, graphite fibers) may originate numerous physical/chemical processes on the polymer and on the carbon phases [27,37]. Such processes may have many effects on material properties, including electrical properties [20,26,28]. 

**Figure 3:**
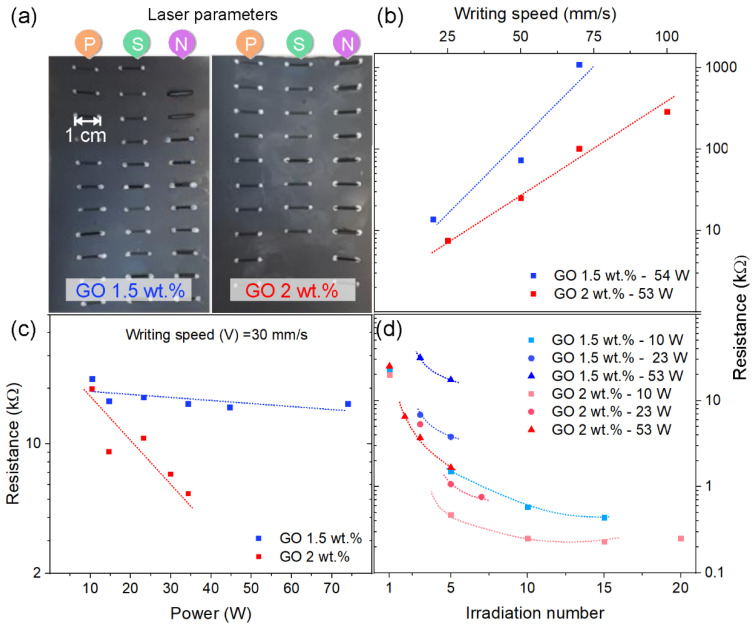
Effect of laser processing parameters (P: laser power; S: writing speed, and N: irradiation number): (**a**) pictures of 10 mm paths obtained on GO-loaded PU layers (GO 1.5 wt.% and GO 2 wt.%); (**b**–**d**) electrical resistance (R) for the 10 mm long paths fabricated by changing different writing speeds, laser powers, and number of irradiations along the same path, respectively.

CO_2_ laser processing was performed on the GO-based PU layer of leathers containing 1.5 and 2 wt.% of GO. A preliminary series of laser treatments was performed on laser-irradiated paths 10 mm in length by adopting different laser parameters, including laser power (P) and scribing speed (S), with the aim of achieving the optimum electrical characteristics (i.e., lower resistance). Repeated irradiations (N) were also performed. Results are shown in Figure 3.

In Figure 3b, the scribing with speeds between 20 and 100 mm/s is illustrated. From this figure, it is clear that by increasing the writing speed, the resistance (R) increased by 1–2 orders of magnitude and that the difference between GO 2 wt.% and 1,5 wt.%-loaded PU is small at low writing speeds (25 mm/s), while it increases significantly at higher speeds (≥75 mm/s). In any case, R is always lower for GO/PU with GO 2 wt.%. In Figure 3c, laser writing with different laser power is illustrated. From this figure, it is clear that by increasing the laser power, resistance R decreases, and that composition plays a crucial role. In fact, the decrease in resistance is significantly larger for PU with GO 2 wt%, even at moderate laser power for a single irradiation path, for writing speed = 30 mm/s, and for the selected laser power intervals. In Figure 3d, the effects of repeated writing along the same 10 mm path and of laser power are illustrated. The process is much more complex if several irradiations are repeated along the same path and considering different writing speeds. Laser irradiation is a very complex phenomenon that can simultaneously produce several thermally activated effects, including melting, vaporization, depolymerization, retro-polymerization, and polymer decomposition processes, as well as ablation and pyrolysis of the polymer and fillers [38,39]. Partial combustion of carbonaceous components is also possible under inert flow conditions when the processing environment is not sealed. Some of these effects may be more favorably directed toward certain processes by some of the process parameters, such as laser power (P) at the sample surface, speed (S), and repetition (N). Although this topic is very broad and goes beyond the scope of this paper, we can conclude that the effect of repeatedly writing along the same path resulted in a drop in electrical resistance R by about two orders of magnitude. The decrease in resistance R was observed to be remarkably higher at the lower laser power and for the first 10–15 repeated irradiations. 

On the basis of all these considerations, it emerged that 2 wt.% GO-loaded PU was the most efficiently processed sample by the laser, and it was subjected to further analyses. In particular, the morphological and structural characteristics of the conductive traces were investigated. 

### 3.3. Morphology and Structure of Laser-Irradiated Regions

**Figure 4:**
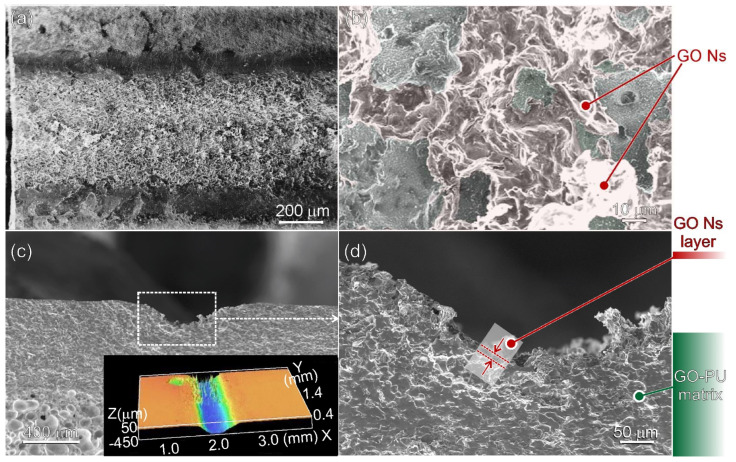
SEM images of a laser-irradiated path: (**a**) low- and (**b**) high-magnification top-view images; (**c**) low- and (**d**) high-magnification cross-sectional views of the GO/PU layer. In the inset of (**c**), an optical 3D profilometry image of the path is illustrated.

The morphology and structure of laser-irradiated leather via single-pass irradiation (N = 1) with 10 W and with a speed of 30 mm/s are shown in Figure 4.

From the top-view SEM images (Figure 4a,b), the effects of the laser beam forming a c.a. 1 mm wide path on the surface of the sample are illustrated (Figure 4a), while from the enlarged SEM image (Figure 4b), it is clear that in the central area of the laser-illuminated path, there are nanosheets with a basal size of 20–40 μm forming a continuous network along the entire path. Two cross-sectional SEM images are shown in Figure 4c,d. By comparing these two images with cross-sectional SEM images of the untreated sample (Figure 1a), it is clear that a groove in the GO/PU layer, c.a. 200–300 μm in depth in its central region, was formed as a consequence of laser treatment (Figure 4c and inset therein). Furthermore, in the region, laser-irradiated leather comprised nanosheets and patches of an irregular shape forming a network that is several microns thick. The appearance outside the groove wall was the same as that generally observed below the percolation threshold for polymer composites containing isolated graphene sheets and platelets [40,41]. 

**Figure 5:**
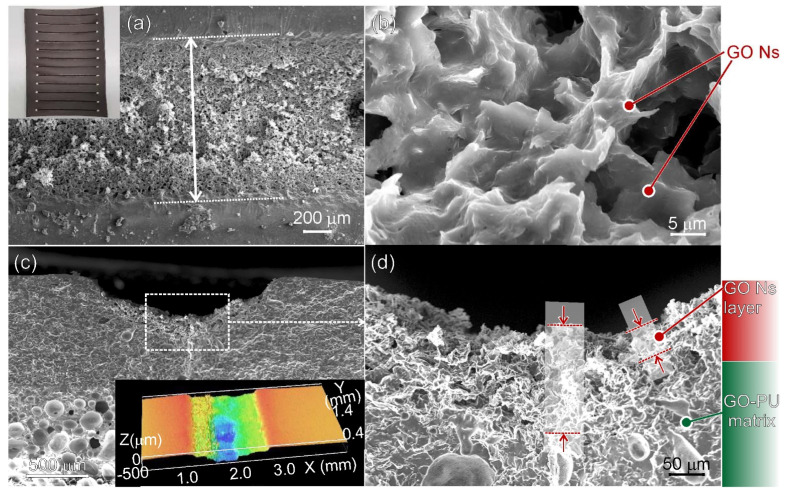
SEM images of a laser-irradiated path: (**a**) low- and (**b**) high-magnification top-view images; (**c**) low- and (**d**) high-magnification cross-sectional views. In the insets of (**a**,**c**), a picture of the irradiated paths and an optical 3D profilometry image of the path are illustrated.

The morphology and structure of laser-irradiated leather via multiple-pass irradiation (N = 15) along the same path by 10 W and with a speed of 30 mm/s are illustrated in Figure 5.

Similar patterns were observed for polymer composites containing nanocarbons irradiated by 5–50W CO_2_ laser power [20,26,27,28,29]. From the top-view SEM images (Figure 5a,b), the effects of laser irradiation forming on the surface of a sample path c.a. 1,8 mm wide are illustrated in Figure 5a, while from the enlarged top-view SEM image (Figure 5b), it was clear that the multiple-pass irradiated path constituted nanosheets with a basal size of 20–40 μm forming a continuous layer along the entire path. Cross-sectional SEM images are shown in Figure 5c,d. By comparing these two SEM images with cross-sectional SEM images obtained by a single-pass irradiation path (Figure 5c,d), it was clear that laser irradiations repeated on the same area caused a deeper groove in the GO/PU layer (Figure 5c and inset therein). Furthermore, the nanosheet layer in the irradiated path was as thick as 40–150 μm, and it was remarkably higher in the central portion of the irradiated region (Figure 5d). Based on the aforementioned results, we can state that (i) electrical resistance R can be measured after laser irradiation, and (ii) R decreased with the thickness and width of this layer consisting of nanosheets. However, nothing was inferred about the nature of the nanosheets after laser treatment by microscopies, and a spectroscopic investigation was performed.

### 3.4. IR and Raman Spectroscopies of the GO-Loaded PU Layer before and after Laser Treatment

**Figure 6:**
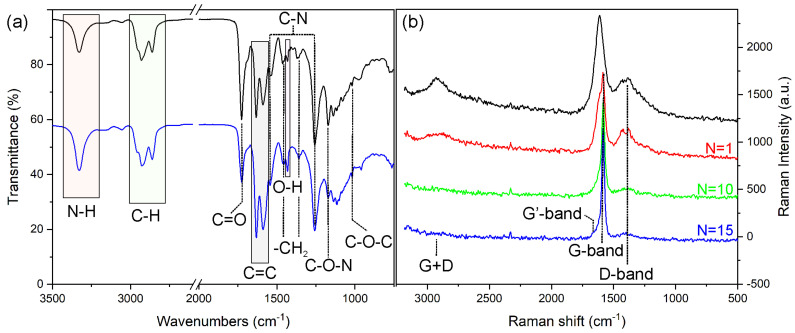
GO/PU top layer: (**a**) ATR spectra before (black line) and after laser irradiation (blue line); (**b**) Raman spectra before (black line) and after 1, 10, and 15 repeated laser treatments (red, green, and blue line, respectively).

The ATR and Raman spectra of the 2 wt.% GO-loaded PU layer before and after laser irradiation are shown in Figure 6a.

The IR spectrum of untreated PU leather (Figure 6a, black line) shows an absorption band at 3326 cm^−1^ and a band envelope at 2958 (shoulder), 2929, and 2861 cm^−1^, which are associated with N–H and aliphatic symmetric/asymmetric CH_2_ stretchings [42]. A band with a maximum at 1727 and shoulder at 1700 cm^−1^ can be assigned to free C=O and H–bonded C–O stretching [31], respectively. Furthermore, absorptions at 1632 and 1590 cm^−1^ indicate the aromatic ring skeleton (C=C) of PU [43]. C–N bondings in PU have distinctive IR bands at 1539 and 1257 cm^−1^ [31]. Additional bands at 1461, 1167, and 1139 cm^−1^ can be assigned with alkoxy C–O, alcoholic (C–OH), and epoxy O–C–O stretching, respectively [36,44]. No adsorption band assigned to the isocyanate region of the prepolymer was found (c.a. 2312 cm^−1^), thus indicating complete PU crosslinking [36]. The IR spectrum of the PU surface on the laser-irradiated path (Figure 6b, blue line) exhibits absorption peaks of different relative intensities at 1725, 1462, 1432, 1359, 1171, and 959 cm^−1^, associated with the O- and N- functional groups, which are indicative of a certain degree of degradation and evolving oligomers [45] induced by localized laser heating [46]. No significative variation of the aromatic ring skeleton bands can be observed (c.a. 1640–1550 cm^−1^), as also reported for PU containing 1–3 wt% graphene [47]. This may be attributed to the fact that the IR spectroscopy of conjugated C–sp^2^ bonding is greatly affected by the presence of both chemical and physical defects, such as polar groups at the surface [48]. To this purpose, Raman spectroscopy was adopted to shed light on the carbon components of composite leather. The Raman spectra of GO/PU before and after laser irradiation are shown in Figure 6b. The main Raman fingerprints of GO/PU at 2925, 1612, 1441, and 1376 cm^−1^ are associated with the symmetric stretching vibration of −CH_2_ [49,50,51], C=C aromatic breathing mode vibrations, bending vibration of −CH_2_, and D-band of GO [50,51], respectively. The D-band is associated with structural disorder, and its intensity is inversely related to crystallinity [49]. There is also a contribution of the G peak (E_2g_ mode) of GO with maximum c.a. 1585 cm^−1^ [50] to the main broad feature. The situation after laser irradiation was remarkably different. The band at 2925 cm^−1^ decreased in intensity, while the band at c.a. 1600 cm^−1^ was eroded, exhibiting a shoulder at 1612 cm^−1^ and a narrow peak at 1585 cm^−1^. 

After further laser treatments (N = 10), the PU features disappeared, and Raman spectra were fully dominated by carbon fingerprints (Figure 6b, green and blue lines) and in accordance with the Raman spectra of PU containing 1–3 wt.% of graphene, whose PU fingerprints decreased in intensity or disappeared with increasing the graphene loading [47]. As for G- and D-fingerprints, the G-band was narrowed and shifted to 1581 cm^−1^, while the D-band decreased in intensity. In addition, the D-band at 1620 cm^−1^, corresponding to an intra-valley resonance with the G-band in the presence of impurities [50] appeared at the increasing laser treatment (N = 15). All these observations indicated that laser treatment was responsible for the remarkable chemical modification of the irradiated path, with partial polymer decomposition and with the formation of reduced-GO (RGO) sheets. The decomposition of such PU was thermally observed from 200 °C, as observed from the TGA plot (Figure 2). On the other hand, the reduction in GO promoted by CO_2_ laser irradiation is in accordance with several studies [46,51,52]. While qualitative identification of Raman spectra is possible, a more quantitative approach including I_G_/I_D_ evaluation is not possible due to the complex band envelope in the Raman spectra before laser irradiation. However, a decreased I_G_/I_D_ with increasing irradiation time can be attributed to the gradual decreased intensity of the D-band. Interestingly, the G- and D-bands together with the absence of second-order fingerprints (2D and D+G bands) were informative of a multilayer structure of reduced-GO sheets that contain a certain degree of structural disorder [51]. Furthermore, according to the SEM images shown in Figure 4 and Figure 5, it was concluded that after laser treatment, the irradiated paths were made of a continuous 3D envelope of reduced-GO sheets. In the following paragraphs, the effects of laser treatment with the resulting formation of reduced-GO paths will be analyzed from an electrical viewpoint.

### 3.5. DC and AC Electrical Properties before and after Laser Processing 

**Figure 7:**
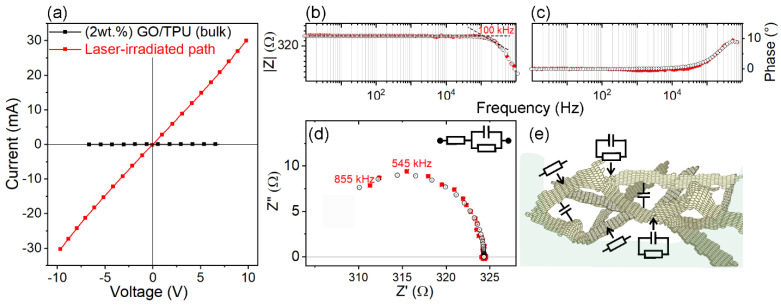
DC and AC electrical properties of a 60 mm conductive laser-irradiated path as obtained by four-probe measurements (electrical measurements by four 10 mm spaced electrodes): (**a**) I–V graph of the conductive track (red curve), as compared to GO/PU polymer composite leather (GO 2 wt.%) (black curve); (**b**–**d**) Bode magnitude and phase and Nyquist plots for the same conductive path (red points) in the 1 Hz–900 kHz frequency range, respectively. EIS-fitted curves (B/N points) are shown for comparison in (**b**–**d**); (**e**) schematic representation describing capacitance (C) and resistance (R) elements of the RGO sheets in the conductive paths after laser irradiation according to frequency responses.

In Figure 7, the DC and AC electrical properties of laser-irradiated paths are illustrated. 

In Figure 7a, DC current–voltage (I–V) properties obtained by four-wire resistance measurements on the laser-irradiated path are compared with the bulk properties of GO/PU polymer composite leather (2 wt.% of GO). In this figure, a linear dependence between the current and the voltage drop within a ±10 V interval is shown as compared to the GO/PU polymer composite leather. It is clear that the laser-irradiated paths were the only electrically conductive regions, while the surface not treated with laser and the bulk of the GO/PU leather were both insulating. It is worth mentioning that the linearity and ohmic behavior suggest the formation of a continuous path, which is usually observed for carbon-based polymer composites under DC measurements over the electrical percolation threshold [4,6,7,53,54]. The laser-irradiated region can be described as a linear resistance component with moderate electrical resistance, which is calculated to be c.a. 320 per 10 mm of track linear length as obtained from the slope of the red curve. No relevant variations between four-probe and two-probe DC resistance measurements were obtained. Together with the confirmation of DC electrical properties, and for a more complete investigation, impedance measurements were also performed in the small-frequency domain to model electrical properties with the structure of the conducting paths and with junctions between different conductive particles.

Figure 7b and c display Bode magnitude and phase shift plots, which illustrate the frequency dependence in the 1 Hz–900 kHz interval for a 60 mm conductive laser-irradiated path (four 10 mm spaced electrodes in the central path region). The AC electrical properties in the lower-frequency domain (Bode magnitude plot, Figure 7b) showed that impedance magnitude |Z| was constant and did not depend on the frequency until a critical value, which was estimated to be about 100 kHz (see the tangent lines’ intersection shown in Figure 7b). Furthermore, the impedance phase angle *(φ)* was near 0° until the critical frequency value (Figure 7c). Taken together, these two simultaneous observations are representative of carbon-based composites with filler concentrations above the percolation threshold (*Φ_c_*) [55,56] and corroborate the fact that conductive paths included not only a resistive component but also a capacitive phase. 

In Figure 7d, a Nyquist diagram in the 1 Hz–900 kHz frequency interval is shown. In this plot, real impedance Z’ versus imaginary impedance Z” for a 10 mm conductive laser-irradiated path is illustrated. Ohmic conduction behavior occurred at the lower frequencies, and capacitive effects were neglected, while the higher frequency domain of the spectrum exhibited a semicircle shape with a Z” maximum at c.a. 545 kHz. In this regard, the contribution at high frequencies of individual capacitive effects, which are frequency-dependent, occurred between the conductive RGO sheets and contributed to the overall impedance from the critical frequency that is calculated to be about 100 kHz. The contribution of such coupling capacitances between conductive sheets was, however, very limited compared to that of compounds with filler concentrations below *Φ_c_* [20,55]. 

The Nyquist (Cole–Cole representation) and the Bode plots were well-matched by an equivalent circuit model consisting in resistance (*R*_1_) in series with additional resistance (*R*_2_) and capacitance (*C*) in parallel (Inset of Figure 7d). A physical interpretation of this equivalent circuit model can be made based on several features. Firstly, the imperfect contact between RGO and electrode materials (i.e., contact resistance, *Rs*) contributes to *R*_1_ together with the intrinsic resistance of RGO along the sheets. Secondly, the overall structure of the RGO sheet scaffold, including sheet proximity and junctions, originates capacitance *C* and contributes to the *R* and *C* elements in parallel, respectively (Figure 7e). Furthermore, high-frequency signals through conventional and unconventional conductors are affected to a certain extent by skin effects (i.e., current density is higher near the surface of the conductor), which alters the operative transport characteristics [57].

### 3.6. Piezoresistive Properties

**Figure 8:**
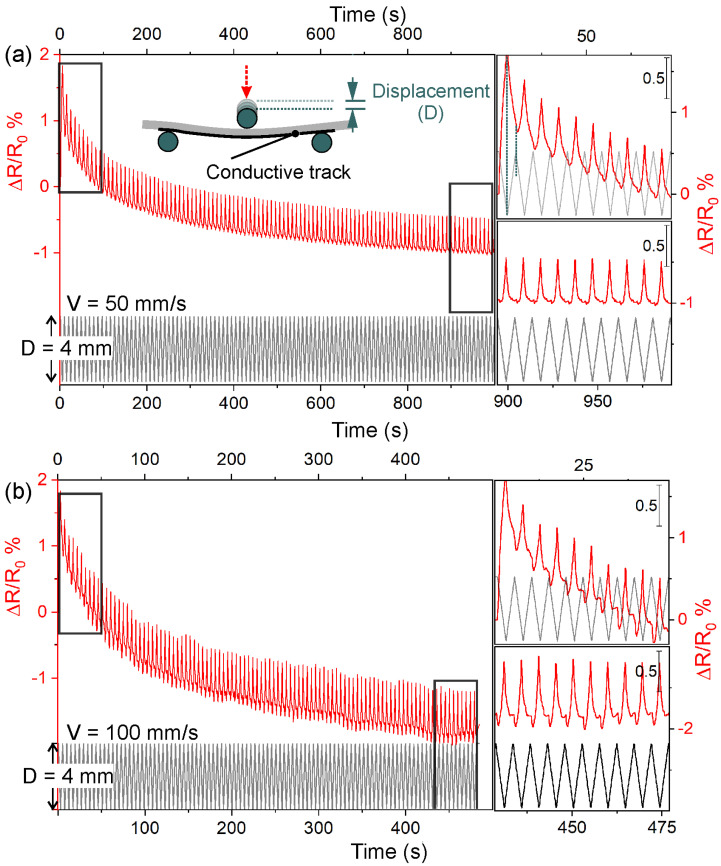
Piezoresistive response of the 60 mm laser-irradiated conductive tracks in the 2 wt.% GO-loaded PU composites measured by the 3-point bending method for 4 mm of displacement for different cycling speeds. Variation of ΔR/R_0_ (red line) for 100 loading/unloading cycles (black line) using (**a**) 50 mm/s and (**b**) 100 mm/s. ΔR/R_0_ variation in the first and last 10 loading/unloading cycles of (**a**,**b**) is shown in the top and bottom insets, respectively.

The piezoresistive properties of GO/PU (2 wt.% GO) after laser irradiation were determined for specimens 20 × 60 mm in size via the three-point-bending method during repeated cycles of displacement (D) of 4 and 5 mm using 50 and 100 mm s^−1^ as cycling speeds. Piezoresistive curves for D = 4 mm and cycling speeds of 50 and 100 mm/s are illustrated for the first 100 cycles (Figure 8a,b).

From these curves, it is clear that ΔR/R_0_ decreased more rapidly in the first 10–20 deformation cycles, and then it progressively become more stable (see top and bottom insets in Figure 8a,b). We can associate this rapid decrease in ΔR/R_0_ with a plastic deformation. Furthermore, the piezoresponse and displacement variation were observed to be synchronous, with ΔR/R_0_ maxima corresponding to the maximum deformations. A broadly similar decreasing trend for the first 100 cycles was observed when the speed was 100 mm s^−1^ (Figure 8b). However, under these conditions, there were significant differences in the definition of piezoresistive characteristics for both the first and the last 10 cycles. Notwithstanding the synchronization between the piezoresistive signal and elongation, the ΔR/R_0_ profile was more asymmetrical and distorted. This asymmetry corresponded with the displacement return. Remarkably, the dimensional recovery of the material was too slow when the speed was 100 mm s^−1^, but this behavior was less than 20 per cent of the piezoresistive signal magnitude observed in each cycle.

**Figure 9:**
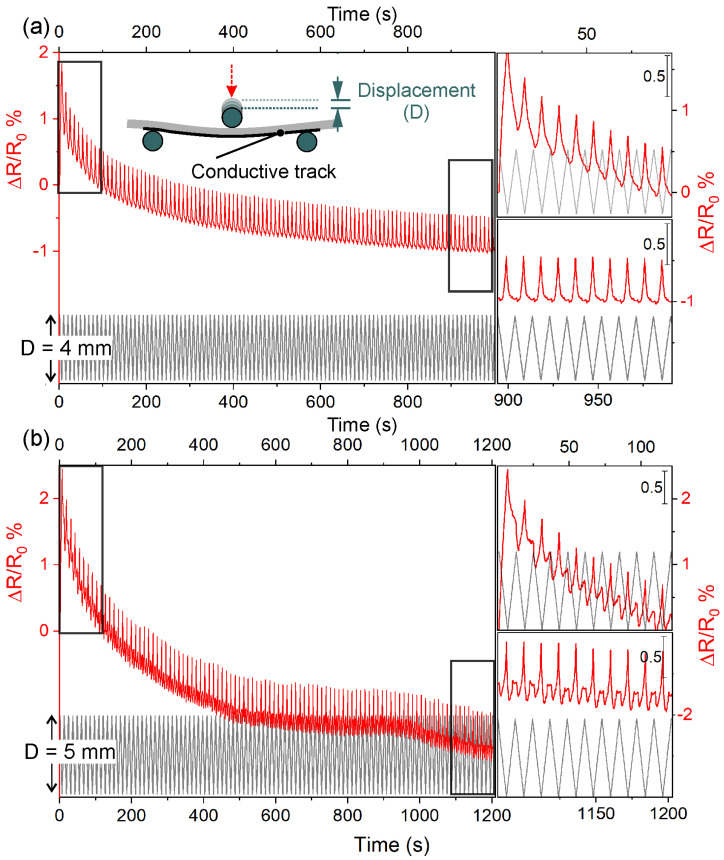
Variation of ΔR/R_0_ (red line) for 100 loading/unloading cycles (black line) using a cycling speed of 50 mm/s under different displacements: (**a**) 4 mm and (**b**) 5 mm. ΔR/R_0_ variation in the first and last 10 loading/unloading cycles of (**a**,**b**) is shown in the top and bottom insets of each plot, respectively.

The piezoresistive curves in the first 100 cycles for D = 4 and 5 mm and cycling speeds of 50 mm/s are compared in Figure 9a,b.

From these curves, it is clear that the higher displacement (5 mm) affected the piezoresponse, which decreased more rapidly in the first 10–20 cycles of deformation but continued to decrease in the last 10 cycles of 100. Although the piezoresponse and displacement variations were still observed to be correlated, the ΔR/R_0_ peaks were observed to be highly asymmetric. In addition to strong cycle asymmetry, there was a second rebound peak corresponding to the displacement return, which was much more defined in the last 10 cycles (Figure 9b, inset at the bottom). It is worth mentioning that the piezoresistive behavior was remarkably affected by the higher deformation, and that during the unloading step, the presence of rebound peaks can be attributed to the rearrangement of the percolation paths of RGO with the formation of new paths and destruction of old RGO sheet networks, as observed by other authors [58,59,60]. Furthermore, a small contribution to piezoresistive response in all these tests, determined by geometric shape variation, cannot be excluded. 

**Figure 10:**
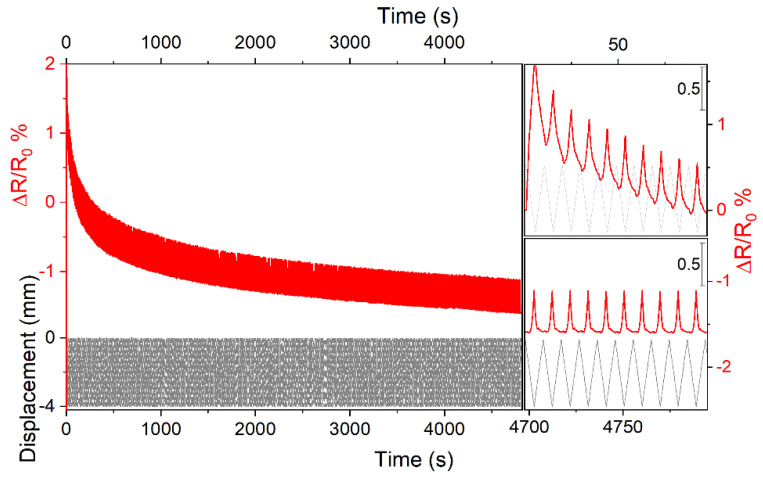
ΔR/R_0_ duration test (red line) for 500 loading/unloading cycles (black line) using a cycling speed of 50 mm/s for a displacement of 4 mm. The ΔR/R_0_ variation in the first and last 10 loading/unloading cycles is shown in the top and bottom insets, respectively.

A duration test was performed to evaluate the piezoresistive response for 500 deformation cycles using a cycling speed of 50 mm/s and a displacement of 4 mm (Figure 10). 

From this figure, it is clear that ΔR/R_0_ rapidly decreased in the first 10–20 deformation cycles due to a plastic deformation. Then, it was more constant (see top and bottom insets in Figure 10) up to 500 cycles. Notwithstanding the synchronization between the piezoresistive signal and displacement, the ΔR/R_0_ profile was a bit asymmetrical. This asymmetry corresponded with the displacement return regions.

## 4. Conclusions

In this study, flexible PU-based leathers containing 1,5 and 2 wt.% GO in the polymer matrix with insulating properties were electrically activated by CO_2_ laser processing to fabricate conductive paths at the surface exposed to the laser beam. By combining different investigation methods, including SEM, optical profilometry, and IR and Raman spectroscopies, the effects of laser irradiation on the GO/PU leather were revealed. Furthermore, DC and AC electrical measurements were adopted to show their potential use under static and dynamic conditions. In more detail, it was shown that under appropriate CO_2_ laser irradiations, GO sheets in the GO/PU layer were locally photoreduced to form entangled RGO sheets, creating an accumulation region at the surface directly exposed to the laser beam. When the laser process was performed along regular paths, these RGO sheets formed electrically conductive wires, which exhibited piezoresistive properties when exposed to mechanical deformation. It was verified that these piezoresistive regions functioned quite linearly when subjected to small bending stresses. The piezoresistive properties were sufficiently effective for stress monitoring and showed durable properties when subjected to repeated bending cycles (N ≥ 500). In summary, laser-activated GO/PU artificial leathers may represent a new generation of metal-free materials for low-current electrical signal transport applications and embedded deformation sensors.

## Data Availability

Not applicable.
